# Utilizing patient data from the veterans administration electronic health record to support web-based clinical decision support: informatics challenges and issues from three clinical domains

**DOI:** 10.1186/s12911-017-0501-x

**Published:** 2017-07-19

**Authors:** Nallakkandi Rajeevan, Kristina M. Niehoff, Peter Charpentier, Forrest L. Levin, Amy Justice, Cynthia A. Brandt, Terri R. Fried, Perry L. Miller

**Affiliations:** 1 VA Connecticut Healthcare System, 950 Campbell Avenue, West Haven, CT 06516 USA; 2 Yale Center for Medical Informatics, Yale University School of Medicine, 300 George Street, Ste 501, New Haven, CT 06511 USA; 3 Department of Medicine, Yale University School of Medicine, 333 Cedar Street, New Haven, CT 06510 USA; 4 Yale University School of Public Health, 60 College Street, New Haven, CT 06520 USA; 5 Department of Emergency Medicine, Yale University School of Medicine, 333 Cedar Street, New Haven, CT 06510 USA; 6 Department of Anesthesiology, Yale University School of Medicine, 333 Cedar Street, New Haven, CT 06510 USA

**Keywords:** Clinical decision support, Electronic health records systems, Biomedical informatics

## Abstract

**Background:**

The US Veterans Administration (VA) has developed a robust and mature computational infrastructure in support of its electronic health record (EHR). Web technology offers a powerful set of tools for structuring clinical decision support (CDS) around clinical care. This paper describes informatics challenges and design issues that were confronted in the process of building three Web-based CDS systems in the context of the VA EHR.

**Methods:**

Over the course of several years, we implemented three Web-based CDS systems that extract patient data from the VA EHR environment to provide patient-specific CDS. These were 1) the VACS (Veterans Aging Cohort Study) Index Calculator which estimates prognosis for HIV+ patients, 2) Neuropath/CDS which assists in the medical management of patients with neuropathic pain, and 3) TRIM (Tool to Reduce Inappropriate Medications) which identifies potentially inappropriate medications in older adults and provides recommendations for improving the medication regimen.

**Results:**

The paper provides an overview of the VA EHR environment and discusses specific informatics issues/challenges that arose in the context of each of the three Web-based CDS systems. We discuss specific informatics methods and provide details of approaches that may be useful within this setting.

**Conclusions:**

Informatics issues and challenges relating to data access and data availability arose because of the particular architecture of the national VA infrastructure and the need to link to that infrastructure from local Web-based CDS systems. Idiosyncrasies of VA patient data, especially the medication data, also posed challenges. Other issues related to specific functional needs of individual CDS systems. The goal of this paper is to describe these issues so that our experience may serve as a useful foundation to assist others who wish to build such systems in the future.

## Background

The US Veterans Health Administration (VHA) electronic health record (EHR) is mature and has been implemented and used at least since 1999 [1]. The VHA has developed a robust and mature computational infrastructure in support of its EHR, including: 1) the Veterans Health Information Systems and Technology Architecture (VistA) which provides backend database support for clinical transactions, 2) the Computerized Patient Record System (CPRS) which provides an interface to VistA, and 3) the VA Corporate Data Warehouse (CDW) which contains national patient data from VistA structured to allow diverse analysis and reporting. To facilitate research community’s access to VA data, while ensuring security and veteran’s privacy, and to provide a computational infrastructure for analysis of the data, the VA Informatics and Computing Infrastructure (VINCI) has also been established https://www.hsrd.research.va.gov/for_researchers/vinci/. VINCI provides medical and Decision Support System (DSS) data in various formats including SAS and SQL. VistA and CPRS currently include a variety of built-in clinical decision support options, including automated alerts and reminders (e.g. Clinical Reminders), structured templates to guide patient management, and online access to clinical guidelines and other clinical reference information.

The three CDS systems we have developed and are described below complement other research projects that are exploring CDS within the VA EHR environment. These include the ATHENA system that provides CDS in the clinical domains of hypertension [2], opioid therapy for chronic, non-cancer pain [3, 4], and previous work at VACHS providing CDS for patients with HIV [5]. An expanding list of applications built around the VA EHR and currently available through VistA can be found at the VA software document library web site (https://www.va.gov/vdl/).

To provide CDS tailored to a patient’s care, such a system must be able to access clinical data and use that data as a basis for patient-specific CDS. For many CDS applications, this interaction needs to take place in real-time, or very close to real-time.

This paper first describes the three Web-based CDS systems we developed and implemented within the VA EHR environment and specific informatics-related challenges and issues that arose in the process of implementing each system. Finally, we also discuss a more general data access problems involved in identifying a patient’s medications, which is an issue that is likely to be confronted in the implementation of many CDS systems. We believe that a discussion of these methods and informatics-related challenges and issues may be of assistance to other groups interested in implementing Web-based CDS within the VA.

## Methods

We developed the following three Web-based CDS systems in support of three research projects based at the VA Connecticut Healthcare System (VACHS):

- The VACS (Veterans Aging Cohort Study) Index Calculator [6] which estimates prognosis for HIV+ patients,
- Neuropath/CDS [7] which assists in the medical management of patients with neuropathic pain,
- TRIM (Tool to Reduce Inappropriate Medications) [8] which identifies potentially inappropriate medications in older adults and provides recommendations for improving the medication regimen.

These systems are integrated into the VA EHR patient data infrastructure as discussed below.

## The VA EHR environment

The components of the VA EHR infrastructure that our three CDS systems are built upon are:

- *VistA*: VistA provides the backbone of the VA EHR. VACHS has its own instance of VistA. VistA stores patient data in a MUMPS data structure that is designed to support efficient real-time patient-specific transactions in support of clinical care. VistA provides a range of Web services, each designed to allow a particular type of patient-specific data to be retrieved, for example, laboratory results, active medications, demographics, etc. All three of our CDS systems use these Web services to retrieve data from VistA in real time.
- *CPRS*: CPRS is the interface to VistA used by clinicians when performing patient care. Two of our CDS systems are activated directly from CPRS via the CPRS Tools menu. For example, to activate the VACS Index Calculator, the user goes to the CPRS Tools menu, selects the “CLINICAL RESOURCE LINKS” submenu, and then selects “VACS Index Calculator”. This results in CPRS linking to the VACS Index Calculator Web site (using its URL web address) and passing the patient’s VistA ID as a parameter. The CDS system then activates a Web-based CDS window on the PC being used to access CPRS.
- *The CDW*: The VA CDW contains national patient data from VistA, updated every night. The CDW infrastructure includes four regional data warehouses (RDWs), which receive data from VistA instances in their region and which in turn update a single national CDW. We obtain CDW data from the RDW for VA Region 4. Whereas VistA is designed to support efficient patient-specific data retrieval, the CDW is a relational SQL Server database and is designed to support data analysis and reporting across multiple patients for which real-time response is not necessary. To make CDW data available for efficient retrieval by a CDS system, we download selected data for all VACHS patients weekly from the RDW. Those data are processed locally (as described below), and placed in a local “staging database”, which is an SQL Server database on the local VACHS network. This design allows relevant selected CDW data to be accessed with real-time response by our CDS systems.

## System descriptions

- *The VACS Calculator:* The VACS Index Calculator was developed as part of the Veterans Aging Cohort Study (VACS). It uses clinical data from a patient who is HIV+ to estimate that patient’s prognosis (5-year mortality), and to allow the user to explore various ways in which that prognosis might be improved. The VACS Index Calculator can be accessed from within the VACHS via CPRS. The patient’s clinical data is obtained automatically from the EHR in real time using the Medical Domain Web Service (MDWS) available from VistA. The VACS Index Calculator can also be accessed from anywhere via the Web (https://medicine.yale.edu/intmed/vacs/) where the user can enter clinical data manually.

**Fig. 1:**
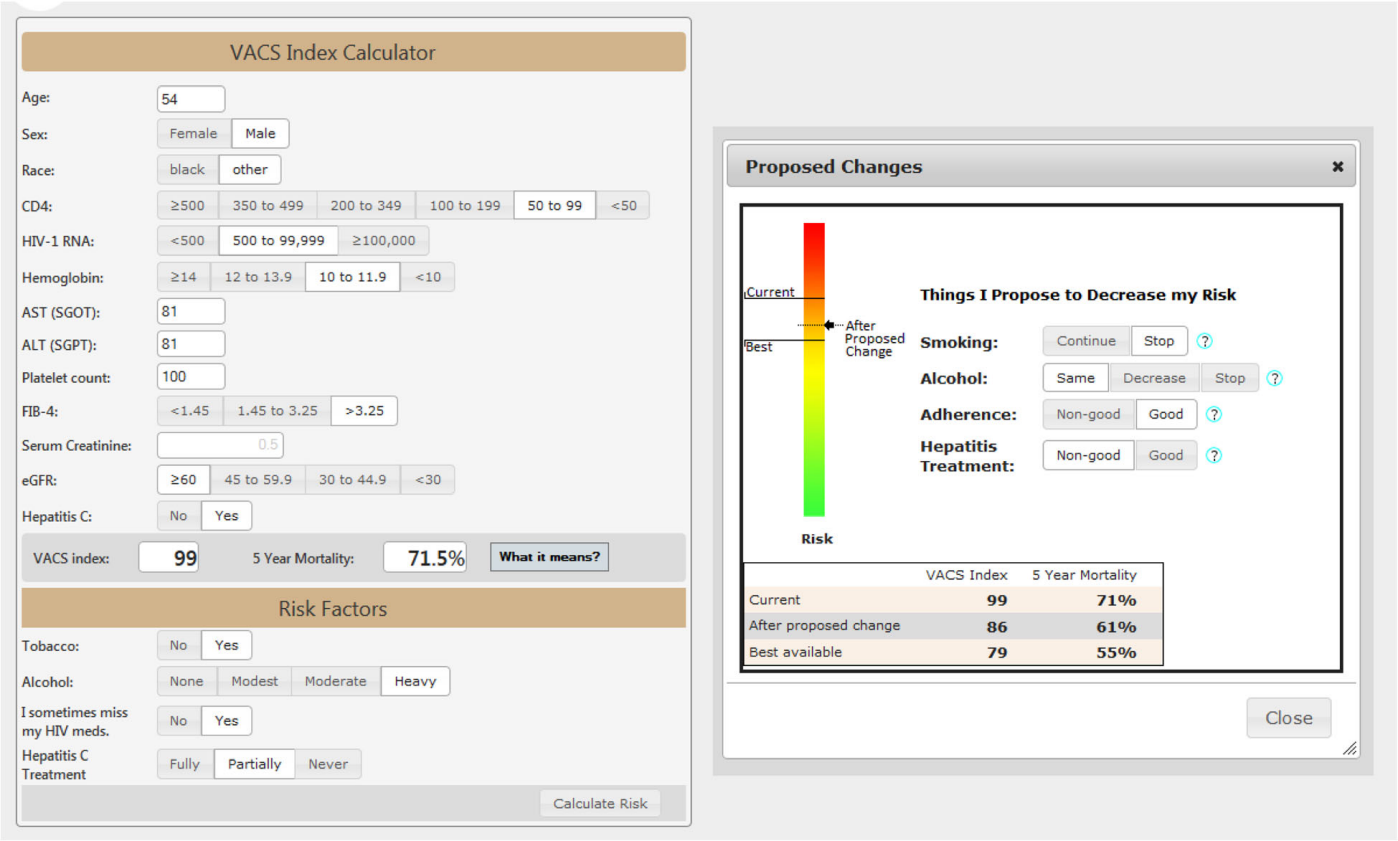
The Web interface of the VACS Index Calculator

Figure 1 shows the system’s Web interface. The main screen, on the left, shows the input data retrieved from VistA, which includes age, sex, race, and a variety of laboratory test results. These data are used to calculate the patient’s current 5-year mortality estimate. At the bottom of that screen, the clinician can enter information about the patient’s smoking, alcohol consumption, HIV drug adherence, and Hepatitis C treatment status (if relevant). Based on this information, the clinician can explore, and demonstrate to the patient, if desired, how the 5-year mortality could change based on behavior changes on the part of the patient.

From an informatics perspective, the VACS Index Calculator is the simplest of the three CDS systems. All the retrieved data are obtained from VistA through MDWS. No data are required from the CDW.

It is important that the user be able to determine how recent each of the laboratory values was. For this, a link is included that can display an expanded view of the test results showing the date when each laboratory value was obtained.

We also allow the data displayed to be changed by the user based, for example, upon other test results from outside the VA. In this regard, it is particularly important to maintain consistency of the values that might be entered in the portions of the screen that allow automatic computation of certain parameters. For example, as shown in Fig. 1, if the test values of Aspartate Aminotransferase (AST), Alanine Aminotransferase (ALT), and Platelet Count are present, these are used to automatically select a range for Fibrosis-4 (Fib-4). The user can change the value of any of these fields and in this case the computed Fib-4 range may change. Alternatively, the user can manually change the range for Fib-4, in which case, any values entered for the three tests are not used and so removed. It required very careful Web programming and testing to handle correctly all combinations of changes the user might iteratively make to these actual and computed values.

The data entered at the bottom of the main screen about tobacco, alcohol, adherence and Hepatitis C, are entered by the clinician. Although we might have attempted to infer some of this information from the EHR, it was felt 1) that this would be technically difficult and not very reliable, and 2) that in any case the important part of this CDS capability was to support and encourage communication and discussion between the clinician and the patient.

- *Neuropath/CDS:* Neuropath/CDS is a pilot system developed as a research project to explore and assess various ways in which CDS for patients with neuropathic pain (NP) might be structured via a Web interface in a clinician-friendly fashion. Neuropath/CDS is no longer active because the research project ended and it would have required frequent updates to keep its clinical knowledge current. Neuropath/CDS was accessed within the VACHS via CPRS with clinical data automatically obtained from the EHR.

**Fig. 2:**
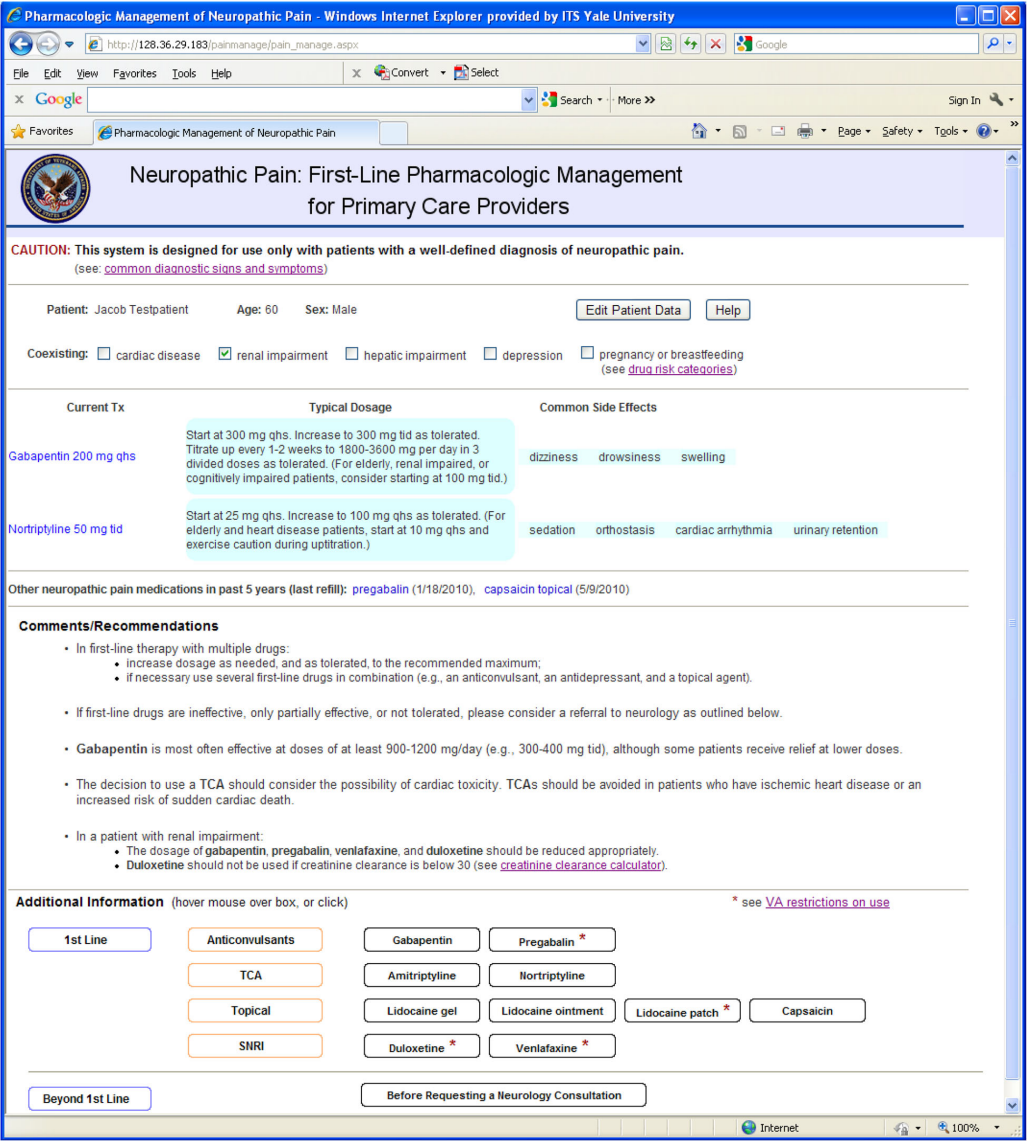
The Web interface of Neuropath/CDS

Figure 2 shows the system’s interface, which includes the following patient-specific information 1) demographic information, 2) the presence or absence of certain diseases, 3) current NP medications, and 4) the last date that other NP medications were prescribed, if any. It then presents comments and recommendations and other information relating to the patient’s NP management.

The demographic information and active medications were obtained using MDWS from VistA. The active medications were obtained from two VistA tables depending on whether they were VA prescribed or non-VA prescribed. (The medication data posed additional challenges, as discussed later in more detail.)

The information about concurrent diseases (based on ICD9 codes) and previously prescribed NP medications was downloaded from the CDW. This information needed to be downloaded for all VACHS patients, so it would be available for any patient selected by the user clinician.

Before this data was placed into the local staging database, it was preprocessed as follows. The ICD9 codes were scanned for codes specifically relevant to the five diseases we were interested in, but the only information stored in the staging database was a flag indicating the presence or absence of each of these diseases. The data on previous medications was searched for any NP medications that had been previously prescribed but were not currently prescribed. Only the names of each such medication found and the date of its most recent prescription were stored in the staging database. This preprocessing facilitated efficient real-time retrieval of this data.

- *TRIM:* The Tool to Reduce Inappropriate Medications (TRIM) was developed as part of a research project to identify older adults at high-risk of receiving potentially problematic medications and to evaluate the medication regimen systematically for problems with both individual medications and the regimen as a whole. Based on information obtained from the EHR, TRIM is designed to assist both the patient and the clinician by providing medication-related recommendations aimed at decreasing the risks of polypharmacy, based largely on the Beers [9] and STOPP [10] criteria.

The TRIM Web interface is not designed to be used directly by the clinician at the point of care. It is used by the research team 1) to identify patients who might be recruited for the clinical research study, 2) to gather data about those patients from the EHR, 3) to accept additional information obtained from chart review and patient interview, 4) to produce a list of all of a patient’s prescribed medications to allow a reconciliation with medications the patient is actually taking, and 5) to produce a set of patient-specific recommendations regarding potentially inappropriate medications and polypharmacy for use by the clinician and a separate set for use by the patient. These recommendations are printed out and handed to the clinician and the patient by a member of the research team.

**Fig. 3:**
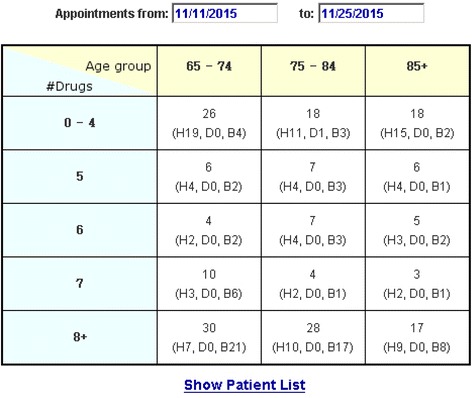
One of TRIM’s Web interfaces, as described in the text

Figure 3 shows the interface used to identify patients who are potentially available to be recruited for the study. Once a week, a list of all patients with primary care appointments scheduled at the West Haven VA is downloaded from the CDW. In addition, the following information is placed into a local VACHS staging database: appointment date, clinic name, patient contact information (address, phone number), age of patient, last 4 digits of their social security number, ICD9 codes, and medications from VistA. To identify patients at high risk according to the presence of polypharmacy and certain chronic conditions, medications are separated into those used in the CDS logic and those that are not (e.g., vitamins, ophthalmic agents, skin creams). ICD9 codes are scanned for the presence or absence of hypertension and diabetes.

Figure 3 shows the number of patients scheduled for clinic appointment within a specified time window (11/11/2015–11/25/2015), organized into subsets based on the patient’s age and number of prescribed drugs of interest. The presence of hypertension and/or diabetes is also noted. For example, the table indicates that there are seven patients scheduled in the age range “75–84” who are taking 6 drugs of interest, and of these 4 have only hypertension (“H4”), none have only diabetes (“D0”), and 3 have *both* hypertension and diabetes (“B3”). Highlighting one or more of these cells and then clicking on “Show Patient List” produces detailed information about each patient including contact information.

The heart of TRIM’s evaluation of medication appropriateness is driven by if-then-else logic. This aspect of the system is beyond the scope of this paper, which focuses on interfacing Web-based CDS to the VA EHR environment.

## Discussion

**Fig. 4:**
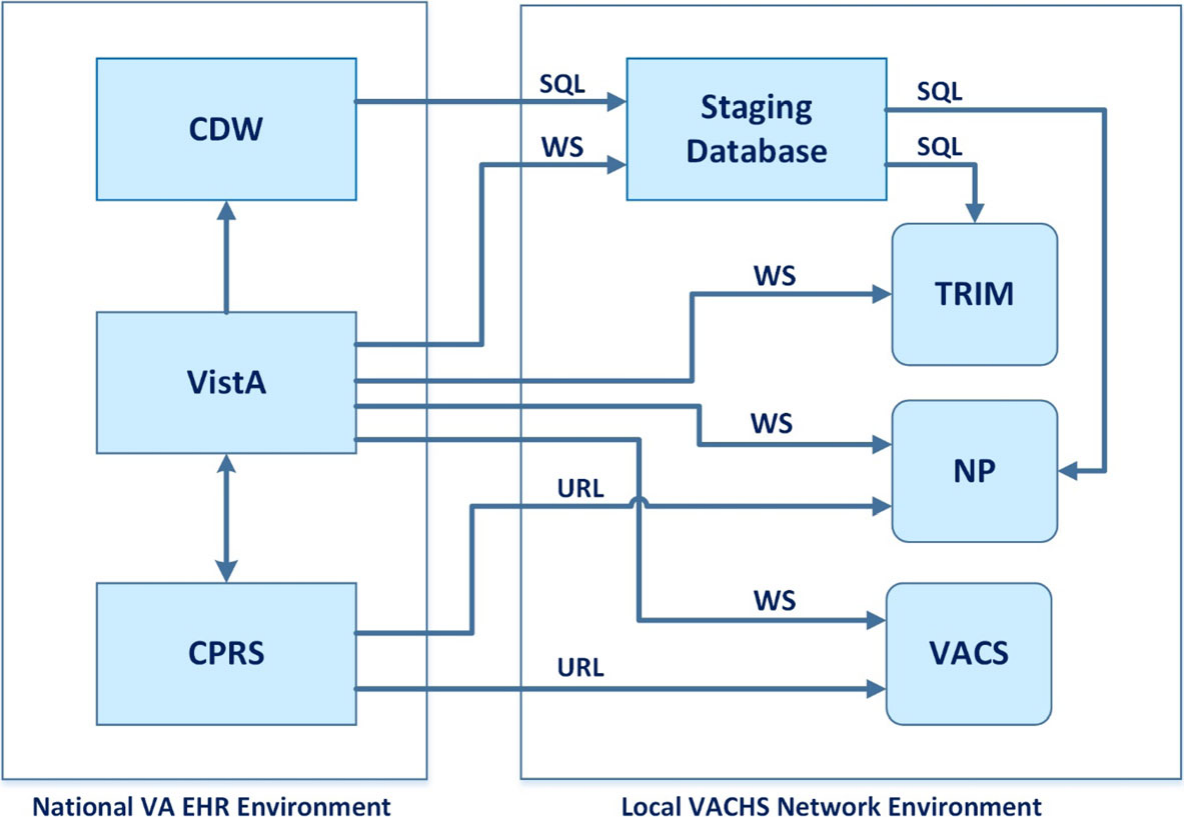
A simplified schematic diagram showing the national VHA EHR environment and the local VACHS network. The diagram illustrates how the three CDS systems fit into this overall structure, as described in the text. The arrows indicate the flow of clinical data. (Abbreviations not used in the text: WS = Web Service; SQL = Structured Query Language; URL = Uniform Resource Locator; NP = Neuropath/CDS; VACS = VACS Index Calculator)

The three web based CDS systems reside in the VACHS network and are linked to the national VA EHR environment as shown in Fig. 4. All these CDS systems use MDWS web service from VistA to get real time patient data for medication, diagnoses, lab tests, or demographics. Data that are not required to be in real time are accessed either from CDW using SQL queries or from VistA using MDWS and are locally stored in a staging SQL Server database. For the VACS Index Calculator, all the retrieved data are obtained from VistA through MDWS, whereas both Neuropath/CDS and TRIM retrieves data from CDW as well as from VistA.

TRIM’s clinical data retrieval from the EHR is almost exclusively focused on medications (aside from identifying hypertension and diabetes using ICD9 codes). An issue specifically related to TRIM involves the need to focus on sets of different drugs for different purposes. To complete medication reconciliation, a list of all prescribed drugs and the class these drugs belong to need to be determined. One challenge involves weeding out many non-drug medical supplies, such as syringes, spacers for aerosol inhalers, lancets for drawing finger-prick blood, and adult diapers, which are all included in the VistA list as medications. To identify patients with polypharmacy, we need to exclude certain medications from the count of number of medications of interest. These include vitamins, cough/cold remedies, over-the-counter drugs, skin creams, ophthalmic agents, and short-term courses of antibiotics. Fortunately, the VA drug formularies have drug classes associated with each drug listed. These drug classes can be used to facilitate identification of the drugs of interest.

## Informatics challenges related to medication data

A major challenge that will confront many CDS systems within the VAEHR involves identifying medications that have been prescribed/filled (currently and/or in the past), including dosage, schedule, and the drug class it belongs to. There are two sources for medication data: VistA (via Web services) and the CDW.

### VistA

VistA data are current (real time). For each prescribed medication, two fields in VistA are of particular relevance, a Drug Name field labeled “name”, and a Drug Schedule field labeled “sig”. Each of these fields contains a text string, which is filled by the prescribing clinician by selecting from a list of values in CPRS. Unfortunately, this list for medication was originally entered as free text that may not exactly correspond to what is in the formulary table Because of this manual free text entry, the contents of these fields can be non-standard and quite variable. Matching this drug name with the corresponding name in the VA drug formularies table was sometimes very problematic. There is also a field for each medication called “dose” in VistA web service, but we found that this field is typically left blank with the dose included in either the name or sig field.

The text string in the Drug Name field is often quite straightforward if it matched with the name in the formulary table, e.g., “PROPRANOLOL 10 MG”. With reasonable frequency, however, it is not, making it difficult to easily identify drug names. The following representative examples illustrate the type of problems we encountered:

METOPROLOL*TWICE DAILY*TARTRATE TAB.

LAMOTRIGINE(LAMICTAL)<SEIZURES > TAB.

NIACIN(SLO-NIACIN) [OTC] TAB, SA.

Determining the dosage and schedule and/or the total daily dose from VistA data is similarly problematical. The examples below illustrate some of these type of problems that we encountered. In each example, the first text string is from the Drug Name field, and the second text string is from the Schedule field.

- “ASPIRIN, ENTERIC COATED(81 MG) TAB, EC”, “162MG MOUTH”
- “WARFARIN NA 5MG TAB”, “TAKE AS DIRECTED BY MOUTH AS INSTRUCTED BY VA ANTICOAGULATION CLINIC (TO PREVENT BLOOD CLOTS)”
- “LISINOPRIL TAB”, “10MG MOUTH DAILY(1000)”
- “FUROSEMIDE TAB”, “20MG MOUTH MONDAY, WEDNESDAY AND FRIDAY”
- “SUMATRIPTAN SUCCINATE 50MG TAB”, “TAKE ONE TABLET BY MOUTH AS NEEDED AT ONSET OF HEADACHE. CAN TAKE AN ADDITIONAL TABLET 1 HOUR LATER IF NEEDED. DO NOT USE MORE THAN TWICE PER WEEK”

These examples are representative of the wide range of variation that presents challenges when attempting to identify dosage and schedule in an automated fashion.

### CDW

The medication data from CDW has been processed and partially cleaned and is typically delayed by one day. Drug names have been extracted and are easily identified. Certain standard dosage/schedule combinations have also been extracted and presented in a standardized format (e.g., “QD”, “QAM”, “BID”, “Q4H”, “Q12H”). For most prescribed/filled medications, however, this processing has not been performed and this field has been left as “NULL”, presumably because of the difficulties discussed above.

## Handling medication data in our CDS systems

The VACS Index Calculator does not utilize medication data, so this was not an issue. Medication data is central to TRIM since TRIM examines all medications prescribed, but TRIM only needs to identify the drug name and its class for its CDS logic processing. Dosage and schedule data are not utilized. Although we use VistA data for the current pilot TRIM implementation (because we were already familiar with accessing and processing VistA data), in retrospect it would have been much simpler if we had used drug name data from the CDW.

In Neuropath/CDS, we were interested 1) in displaying the NP drugs and doses that were prescribed and 2) in calculating the total daily dose (which was tested in the system’s CDS logic). We also needed the data to be real time. As a result, we had to use VistA data. Fortunately, we were only interested in a very restricted set of medications related to NP, which simplified the process of identifying the drug name.

The total daily dosage of a prescribed drug is calculated from its dose, unit, and schedule. These values are determined from the Drug Name and Sig fields using regular expression matching. For example, the following regular expression is used to identify dosage, scanning for strings like “32.5 MG”, “18MCG”, and “(1.5 meq)”.

“[(]?[\d]*[.]?[\d]+[\s]*([mM][gG]|[mM][cC][gG]|[mM][eE][qQ])[)]?”

Similarly, the regular expression below detects common patterns for schedule such as, “bedtime”, “once daily”, and “four times a day”, etc. These text strings are then mapped to prescription abbreviations, e.g., “QHS”, “QD”, and “QID”.

“(bedtime|once[\s]*daily|twice[\s]*daily|three[\s]*times[\s]*a[\s]*day|four[\s]*times[\s]*a[\s]*day)”

For example, if the Drug Name field contains “GABAPENTIN 300 MG, CAP” and the Sig field contains “TAKE ONE CAPSULE BY MOUTH THREE TIMES A DAY”, then 1) we detect 300 as dose and MG as the unit from the Drug Name field, 2) “THREE TIMES A DAY” is mapped to “tid” from the Sig field, and we therefore calculate the total daily dosage as 900 MG.

If this processing is not able to be completed successfully (i.e., total daily dose is not able to be determined), Neuropath/CDS provides Web link labeled “sig” that will display the unprocessed Drug Name and Sig text fields on the user’s Web screen, so the user can view these values. In this case, however, Neuropath/CDS is unable to execute any decision-making rules that depend upon total daily dose.

Our extensive regular expression logic was updated regularly as new patterns for either the dose or the schedule were detected. New unusual and anomalous patterns were periodically encountered. We estimate that this happened considerably less than 10% of the time. As web services provided by VistA are improving regularly, we expect that the percentage of all drug names along with their dosage and schedule values that cannot be automatically decoded will also improve over time.

## Conclusions

This paper describes informatics issues and challenges that arose in the process of implementing three Web-based CDS systems within the VA EHR environment.

- A number of design issues relating to data access and data availability arose because of the particular architecture of the national VA infrastructure and the need to link to that infrastructure from local Web-based CDS systems.
- Several issues arose because of the idiosyncrasies of VA patient data, with medication data posing particular challenges.
- Several issues relate to specific functional needs of individual CDS systems.
- The evolving nature of medicine and of the IT infrastructure resulted in the need to update periodically various components of the approach.

One set of issues that we did not confront involves the use of natural language processing (NLP) to extract information from unstructured textual data. NLP offers the potential to make a wide range of unstructured clinical data available for CDS, as well as a host of challenges.

The functional needs of future CDS systems will likely expose additional issues that need to be confronted. The goal of this paper is to describe specific issues that arose in the context of building Web-based CDS systems in three clinical domains, in the hope that this experience may serve as a useful foundation to assist others who wish to build such systems in the future.

## Abbreviations

ALT: Alanine aminotransferase
AST: Aspartate aminotransferase
CDS: Clinical decision support
CDW: Corporate data warehouse
CPRS: Computerized patient record system
EHR: Electronic health record
Fib-4: Fibrosis-4
HIV: Human immunodeficiency virus
ICD: International classification of diseases
ID: Identifier
IT: Information technology
NLP: Natural language processing
NP: Neuropathic pain
SQL: Structured query language
TRIM: Tool for removal of inappropriate medications
URL: Uniform resource locator
VA: Veterans Administration
VACHS: Veterans Administration Connecticut Healthcare System
VACS: Veterans aging cohort study
VHA: Veterans health administration
VistA: Veterans Health Information Systems and Technology Architecture
WS: Web Service
